# Evaluation of a Parsimonious COVID-19 Outbreak Prediction Model: Heuristic Modeling Approach Using Publicly Available Data Sets

**DOI:** 10.2196/28812

**Published:** 2021-07-26

**Authors:** Agrayan K Gupta, Shaun J Grannis, Suranga N Kasthurirathne

**Affiliations:** 1 Indiana University Bloomington, IN United States; 2 Center for Biomedical Informatics Regenstrief Institute Indianapolis, IN United States; 3 School of Medicine Indiana University Indianapolis, IN United States

**Keywords:** coronavirus, COVID-19, emerging outbreak, modeling disease outbreak, precision public health, predictive modeling

## Abstract

**Background:**

The COVID-19 pandemic has changed public health policies and human and community behaviors through lockdowns and mandates. Governments are rapidly evolving policies to increase hospital capacity and supply personal protective equipment and other equipment to mitigate disease spread in affected regions. Current models that predict COVID-19 case counts and spread are complex by nature and offer limited explainability and generalizability. This has highlighted the need for accurate and robust outbreak prediction models that balance model parsimony and performance.

**Objective:**

We sought to leverage readily accessible data sets extracted from multiple states to train and evaluate a parsimonious predictive model capable of identifying county-level risk of COVID-19 outbreaks on a day-to-day basis.

**Methods:**

Our modeling approach leveraged the following data inputs: COVID-19 case counts per county per day and county populations. We developed an outbreak gold standard across California, Indiana, and Iowa. The model utilized a per capita running 7-day sum of the case counts per county per day and the mean cumulative case count to develop baseline values. The model was trained with data recorded between March 1 and August 31, 2020, and tested on data recorded between September 1 and October 31, 2020.

**Results:**

The model reported sensitivities of 81%, 92%, and 90% for California, Indiana, and Iowa, respectively. The precision in each state was above 85% while specificity and accuracy scores were generally >95%.

**Conclusions:**

Our parsimonious model provides a generalizable and simple alternative approach to outbreak prediction. This methodology can be applied to diverse regions to help state officials and hospitals with resource allocation and to guide risk management, community education, and mitigation strategies.

## Introduction

### Background

The COVID-19 pandemic has impacted the health and well-being of individuals, communities, and economies worldwide at a hitherto unprecedented scale [1-3]. On March 11, 2020, the World Health Organization declared COVID-19 a pandemic with over 118,000 confirmed cases and 4291 deaths in over 114 countries [4]. To date, the pandemic has resulted in over 170 million confirmed cases, with over 3.5 million deaths globally [5]. In the United States, 33 million people have had COVID-19 and more than 600,000 lives have been lost [5].

At the height of the pandemic, waves of viral outbreaks placed health systems across the globe under extended strain, leading to shortages in hospital beds, personal protective equipment, and health care personnel, which caused significant disruptions to health care delivery and loss of life [2,6]. Experts have estimated the cumulative financial costs of the COVID-19 pandemic related to lost output and health reduction at US $16 trillion, or approximately 90% of the annual gross domestic product of the United States [7].

In contrast with historical pandemics, the availability of public and population health information systems has enabled researchers to collaborate on many research activities in response to COVID-19 [8,9]. Since the onset of the pandemic, data scientists have collaborated with governmental organizations to create various public-facing COVID-19 dashboards that provide easy access to descriptive statistics and other metrics [10,11]. Information on COVID-19–related mortality, utilization of health care resources, and recovery has been crucial in increasing situational awareness to inform ongoing pandemic response efforts across communities [12,13].

Most recently, COVID-19 infection rates have started to decrease in response to increased vaccination and efforts in public education [14,15]. To date, 40% of the population of the United States is fully vaccinated [16]. These improvements have led to an interest in relaxing or revoking various restrictions enforced at the state and county levels. While important to the well-being of both communities and economies, such decisions may be dangerous if undertaken without adequate preplanning and awareness of potential risks. As such, effective identification of potential outbreaks of COVID-19 offers the ability to inform decision-makers across governmental and public health sectors on how to resume normal day-to-day activities in their communities and deploy limited human and treatment resources to where they are most needed [17].

Previous studies have demonstrated the potential to apply analytical models to identify potential outbreaks in response to other diseases [18]. However, these methods rely on large, complex data sets extracted from a specific health system or region [19-21]. Such data sets may be challenging and time-consuming to collect, leading to delays in generating timely predictions. Further, models trained using locale-specific data sets may not be generalizable across other locations [22], hindering the potential of reusing such models across other patient populations and regions. A variety of models are trained using complex algorithmic approaches such as neural networks and deep learning models. Such machine learning approaches may yield superior results but fail to achieve widespread acceptance [23] owing to challenges in explainability and interpretation [24].

In contrast, a less complex modeling approach that uses a subset of easily obtainable key elements widely captured across broad geographic regions may be less challenging to develop. Further, such models may also deliver adequate predictive performance without sacrificing explainability and interpretability. Such parsimonious models may also present less risk of overfitting on training data sets, thus allowing for greater generalizability [25].

### Objective

We seek to leverage various readily accessible data sets extracted from multiple states to train and evaluate a parsimonious predictive model capable of identifying the county-level risk of COVID-19 outbreaks on a day-to-day basis.

## Methods

### Methods Overview

We selected 3 states for our efforts in COVID-19 outbreak prediction modeling: California, Indiana, and Iowa. These states were selected on the basis of geographical factors, governmental regulations, and availability of data sets for public use. For example, Indiana and Iowa are similar in the number of counties and total populations [26]. In contrast, California represented a more populous, urbanized state [26]. We also considered the general completeness of reporting, the quality of basic COVID-19 data sources, and the accuracy of state tracking systems [27].

### Data Extraction and Cleaning

For each state, we extracted a variety of county-level data elements captured daily between March 1 and October 31, 2020. Data for Indiana were obtained from the Indiana State Department of Health, while data for Iowa and California were obtained from the New York Times web-based repository [5,28]. We selected March 1, 2020, as a start date as most states began collecting COVID-19 data at this time. October 31, 2020, marked the end of our analysis time period. Each data set was organized by county, state, and date reported using R [29]. Several errors or omissions in the data sets were addressed as follows: days with negative case counts were changed to 0 and a county labeled as “unknown” reported by Iowa and California were removed from further evaluation.

### Preparation of a Gold Standard

We created a gold standard indicating if each county under study was in an outbreak on any particular day. A human expert reviewer created the gold standard by assigning an outbreak label (with responses of “yes” or “no”) to each county or date combination, considering the following criteria:

1. How do case counts trend in each county? Is there a general baseline of cases over time?
2. How large is the county’s population size (counties with more people report more cases)?
3. Duration of the outbreak to assign a binary indicator of “outbreak detected” or “outbreak not detected” to each day and county.

Based on our approach, a county could have multiple outbreaks over time. Outbreaks lasted a minimum of 3 days to account for testing lags as data were not always reported on the same day, especially during the initial phases of the pandemic [30]. Furthermore, lower case counts at the end of an outbreak and on weekends owing to the closure of testing centers were also considered using 7-day average metrics.

### Model Building

We created a heuristic outbreak prediction model using the training data sets obtained from all 3 states and evaluated its performance across the holdout test data sets and the gold standard. For each county, data collected between March 1 and August 31, 2020, were considered the training data set, while data collected between September 1 and October 31, 2020, were considered the test data set. As a preliminary step toward model development, we considered features used in other common models, including the susceptible-infected-recovered epidemic [31] and time delay [32] models, severity of lockdown measures [33], cumulative cases (both reported and not reported) [34], and daily test reports [35]. Furthermore, predictive models for infectious diseases, such as susceptible-infected-recovered models, provide guidance on disease transmission and outbreak causation. The State of Wisconsin’s COVID-19 dashboard used a Case Rate metric defined as a per capita running 7-day sum of the case counts per county per day [36]. Case Rate standardizes COVID-19 severity across counties of differing populations while also accounting for data lags and providing insight on transmission. We plotted Case Rate vs Indiana county populations to generate a general trendline that could differentiate between “outbreak detected” or “outbreak not detected” days. Our logarithmic graph semiaccurately depicted a horizontal line that separated outbreak days. The following steps were undertaken to leverage and apply the trendline results on states and counties with various populations.

**Figure 1:**
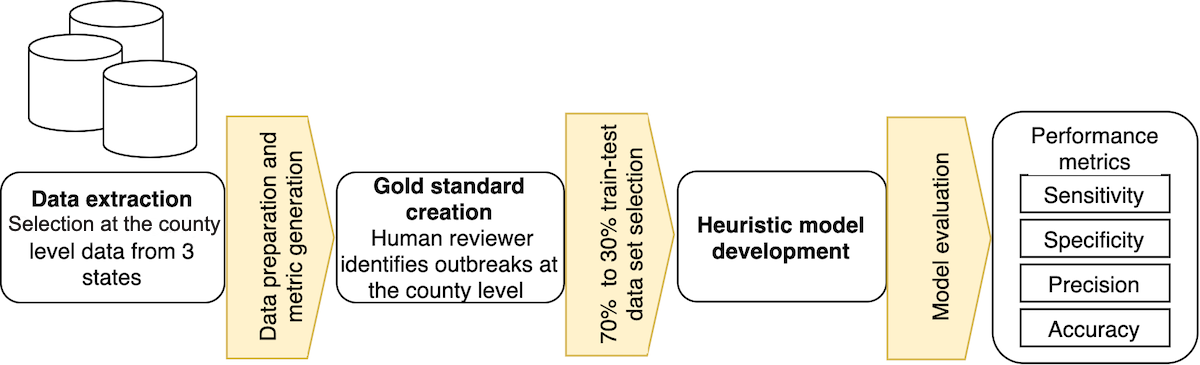
Flow diagram providing an overview of the study methodology.

We started building the model by dividing counties on the basis of population size, initially at 100,000 population intervals. Since Case Rate is more sensitive to less populated counties, we added intervals for counties with less than 100,000 people. Each population interval was allocated an assigned Case Rate baseline value that served as a binary indicator for outbreak determination. We implemented a criterion where an outbreak was underway in counties if they were 4 SDs above the cumulative case count mean to account for data lag. As depicted in the system flow diagram (Figure 1), we established these parameter values and trained the model rules with the training data sets (data reported between March 1 and August 31, 2020). The train-to-test partition was approximately 71% to 29%, respectively, which is close to optimal for large data sets [37]. Then, the model was tested against the gold standard with the test data sets (data reported between September 1 and October 31, 2020). Figure 1 shows a flow diagram depicting our study approach.

## Results

### Results Overview

We collected data on a total of 249 counties from across all 3 states. Table 1 presents descriptive statistics for each state, including the number of counties, population sizes, and urbanization to highlight each state’s fundamental differences [26]. Previous studies have identified multiple factors in determining urban vs rural areas, including the total population, population density, and commuting flow [38].

Indiana and Iowa have similar county population distributions, with both having a majority of counties with less than 100,000 people. However, Indiana has more midsized counties with its largest county having almost 1 million people, while California has several counties having populations of more than 1 million people. Moreover, California has the highest percentage of the urban population (94.95%), with Indiana (72.44%) and Iowa (64.02%) far behind.

**Figure 2:**
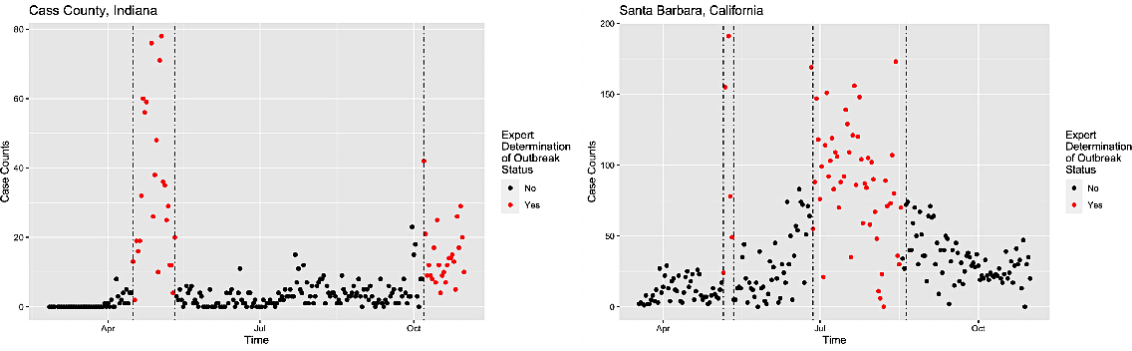
Visualization of the COVID-19 case counts in Cass County, Indiana, and Santa Barbara County, California, between March 1 and October 31, 2020. Outbreak days are indicated in red, and normal days are indicated in black.

Figure 2 provides an example visualization of outbreak determination in Cass County, Indiana, and Santa Barbara County, California, for gold standard preparation. Cass and Santa Barbara counties have populations of 37,689 and 446,499 people, respectively [26].

**Table 1:** State and county population sizes and population statistics based on census counts.

Census counts			Indiana		Iowa		California
**County-level statistics**							
	Counties, n	92		99		58	
	Counties where population is <100,000 people, n	75		93		23	
	Counties where the population is ≥100,000 and <500,000 people, n	16		6		19	
	Counties where the population is ≥500,000 and <1,000,000 people, n	1		0		7	
	Counties where the population is >1,000,000 people, n	0		0		9	
	Population of the smallest county (people), n	5875		3602		1129	
	Population of the largest county (people), n	964,582		490,161		10,039,107	
	Urban population, %	72.44		64.02		94.95	
	Household income (US $), median	59,892		68,718		70,489	
Case count per day, mean (SD)			7.98 (21.02)		6.18 (15.75)		70.33 (231.86)

Table 2 shows the prevalence of the number of outbreaks and their durations in each state over the training, test, and total time periods. In Indiana and Iowa, the number of outbreaks doubled from the training to the test date range, despite the training data set being almost 3-fold as large as the test data set. Furthermore, the percentage of days in an outbreak between the training and test ranges quadrupled to 22.6% and 20.1% for Indiana and Iowa, respectively. The percentage of outbreak days in California remained relatively stable, while the average outbreak duration decreased from 47 days to 19 days. Because counties were our unit of analysis and since California had fewer and more populated counties than Indiana or Iowa, we believe that these factors contributed to the reduced number of outbreaks in California.

**Table 2:** COVID-19 outbreak prevalence descriptors from the gold standard. Indiana, Iowa, and California data sets were divided by training (March 1 to August 31, 2020), test (September 1 to October 31, 2020), and total (March 1 to October 31, 2020) date ranges to characterize the outbreak periods.

Prevalence descriptors	Indiana				Iowa				California		
	Training	Test	Total	Training		Test	Total	Training		Test	Total
Outbreaks, n	26	65	83	43		85	114	35		26	40
Outbreak duration (days), mean	25.00	19.18	22.86	18.18		14.62	15.79	47.29		19.31	53.92
Total outbreak days, n	650	1247	1897	727		1199	1926	1655		502	2157
Outbreak days, %	3.86	22.59	8.45	4.01		20.15	7.97	15.59		14.43	5.24

### Model Rules

Using the aforementioned data sets, we developed model parameters to predict COVID-19 outbreaks.

**Figure 3:**
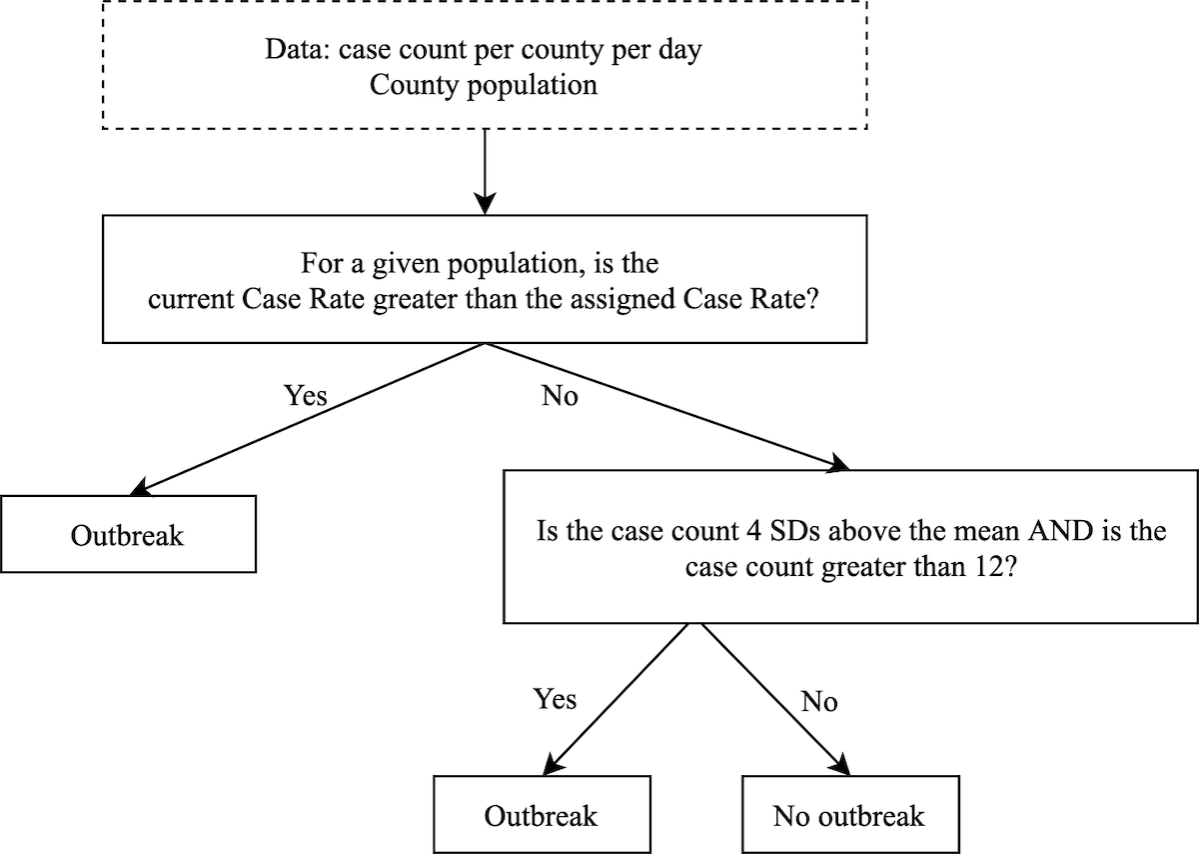
Decision-making process of our heuristic model.

Figure 3 shows a top-down decision tree for our model’s behavior. Rules and the assigned case rate associated with each population band used in the decision-making process are further outlined in Multimedia Appendix 1. As shown in Figure 3, the heuristic model determined that counties experienced an outbreak through the following methods:

1. For the specified population band, a county’s case rate on a given day was greater than the minimum case rate assigned to that population.
2. The county’s case count on a specific day was greater than 12 and was 4 SDs above the rolling mean county-level COVID-19 case count.
3. If a county met either requirement on a specific day, that county was considered to be “in outbreak.”

By combining these rules with the previously developed gold standard, a confusion matrix was utilized to analyze the model’s performance.

Table 3 shows the results of the confusion matrix when the prediction model was applied to the curated gold standard during the test date range from September 1 to October 31, 2020. Sensitivity is the proportion of correctly identified positives, while specificity is the proportion of correctly identified negatives. All 4 key confusion matrix statistics—sensitivity, specificity, precision, and accuracy—were above 80% in each state during the test range. Model specificity and accuracy were >94% for each state. This was attributed to most days being classified as true negatives, which are fundamentally more straightforward to detect than true positives. Model sensitivity for Indiana and Iowa was 10% greater than that of California. However, their precision was 11% and 7% lower, respectively. For Indiana and Iowa, this implies that the model computed fewer false-negative readings, which could be attributed to having increasingly prolonged outbreaks (Table 1). California’s higher precision but lower sensitivity implies that the model was more precise in predicting when outbreaks occurred but less successful in capturing all outbreaks.

**Table 3:** Results of the model with the test data set relative to the gold standard.

Performance parameters	Indiana	Iowa	California
Test date range	September 1 to October 31, 2020	September 1 to October 31, 2020	September 1 to October 31, 2020
Sensitivity, %	92.33	90.05	80.86
Specificity, %	95.56	97.40	99.57
Precision, %	85.04	89.83	96.96
Accuracy, %	94.86	95.91	96.85

## Discussion

### Principal Findings

Our efforts resulted in the development of a heuristic model capable of detecting COVID-19 outbreaks, with predictive measures between 80% and 99%. The model had sensitivities of 92%, 90%, and 81% for Indiana, Iowa, and California, respectively. This indicates that the model was capable of identifying a clear majority of outbreaks across each state. The model also reported precision scores of 85%, 89%, and 96% for Indiana, Iowa, and California, respectively, which indicated that most positive predictions made by the model were accurate. These performance metrics indicate that the model is fit for use in real-life settings. Additionally, the training and test periods displayed distinct outbreak characteristics owing to the increased spread of COVID-19.

These performance metrics are also notable, considering that the prevalence of outbreaks in training data sets was considerably low and could have resulted in weak predictive models had we used more traditional classification-based modeling approaches, which significantly underperform when trained using unbalanced data sets [39,40]. As the pandemic progressed, each state attempted to enhance their data reporting systems. As described by Khakharia et al [41], some regions reported sudden and significant changes in case counts, making it difficult for models to forecast future cases. Though outbreaks are not fundamentally different, the training and test data sets can be characterized separately. Despite the test range being shorter, Indiana and Iowa both reported twice as many outbreaks during the test period. This can be attributed to the second wave of COVID-19 that occurred during the test period as schools resumed, governors relaxed state lockdown laws, and people returned to work [42]. For example, California was one of the last states to begin lifting restrictions in midsized and large counties, which may have contributed to relatively fewer outbreaks than those in Indiana and Iowa [43,44]. Thus, counties re-entered or for the first time realized outbreak periods during the test period.

California remains a state of interest owing to the characterization of its outbreaks as well as predictive performance results on the holdout test data set. Unlike Indiana and Iowa, California has several counties with populations of over 1,000,000 people; furthermore, it was the only state with fewer outbreaks and a low percentage of outbreak days between the training and test periods. The California model revealed a significantly lower sensitivity but higher precision. Thus, to Indiana and Iowa, the California model captured proportionally fewer outbreaks but predicted the subset with greater precision.

This parsimonious prediction model is easily replicable in other states, as it only utilizes county population and COVID-19 cases per day per county data. States can detect and predict outbreaks with high accuracy by following the model’s rules. Current outbreak prediction approaches are based on machine learning algorithms. Though they generally have very high accuracies, these models incorporate a variety of data points and can overfit models [22]. The heuristic model’s data simplicity enables it to be easily implemented in other regions, especially those with limited reported systems. It is also an understandable and accurate method to relay a county’s current state of COVID-19 to the general public, who are not as informed in health metrics. In addition to public and internal communication, forecasting models can be applied to aid in outbreak preparation and community mitigation methods [45].

In addition to a high-performing heuristic model, our efforts also led to the development of a well-curated gold standard data set consisting of outbreak status for each county on a day-to-day basis. This data set is shown in Multimedia Appendix 2 to facilitate additional studies on this important issue.

### Limitations

Our study was impacted by limitations in data collection systems currently deployed by each state. The inconsistency of data reporting presented a significant systematic challenge for model building activities. For instance, states closed most COVID-19 testing centers on weekends, which led to lower case count values on Saturdays and Sundays. Further, many states did not publish most of their own COVID-19 data, which led us to obtain data on cases per day per county from the New York Times instead of a state’s Department of Health, the latter being more accurate. The New York Times would retroactively change case data, making it more unreliable since there were days with negative values.

The lack of previous studies on curating gold standards on disease outbreaks also presents limitations. With no industry standard on defining an outbreak, we created the gold standard on the basis of intuition and the aforementioned specified criteria. Therefore, this process could have been subject to potential confounders, which may have influenced our model’s results. Furthermore, the rule-based model approach is subject to several limitations. Since the model incorporated a 7-day moving Case Rate, there was a lag at the tails of outbreaks as the increased case counts were not initially detected. Even with a parsimonious approach, the parameters derived from our results can greatly differ when applied to other regions. This uncertainty, resulting from parameters, social mandates, and vaccination, is a feature of any prediction model. We helped lessen this uncertainty through our generalizable approach demonstrated in diverse states.

### Future Prospects

The ongoing COVID-19 pandemic has led most major institutions to allocate tremendous resources for its resolution. The model would benefit from a larger sample size of US states, and possibly regions worldwide, to test its generalizability on a more expansive scale. Additionally, we could expand the model’s data range for the third wave of cases and as the COVID-19 vaccine is distributed to a majority of the population, to determine its functionality beyond the scope of this study. Our results could also be translated to provide a clearer epidemiological outlook of diseases. Since the model can predict outbreaks with high accuracy, it could be tested on historical COVID-19 data to determine when most outbreaks occurred easily in a particular region. Moreover, trends and patterns were found across outbreaks among various factors such as lockdown policies, air pollution levels, and civilian obedience. Understanding the causes of outbreaks presents interesting findings related to public policy adaptation in current and future situations.

### Conclusions

This study presents an accurate, generalizable, and explainable COVID-19 outbreak prediction model. The model reported sensitivity scores of >90% in Indiana and Iowa and >80% in California. Furthermore, model specificity and accuracy scores were >94% in every state. These results, coupled with the minimal data inputs required, creates an explainable and easy-to-implement model that governments and policymakers can utilize to assess COVID-19 severity across diverse geographic regions. Future studies are required to test the model in other states and countries by using more recent data. Moreover, the model should be used to identify outbreaks to investigate correlations among external factors such as socioeconomic risks, air pollution, county-level laws, and outbreak development.

## Appendix

Multimedia Appendix 1 Population bands with their respective minimum case rates.

Multimedia Appendix 2 Outbreak gold standard for each county in California, Indiana, and Iowa per day.
